# Optogenetic Stimulation of Lateral Amygdala Input to Posterior Piriform Cortex Modulates Single-Unit and Ensemble Odor Processing

**DOI:** 10.3389/fncir.2015.00081

**Published:** 2015-12-21

**Authors:** Benjamin Sadrian, Donald A. Wilson

**Affiliations:** ^1^Department of Child and Adolescent Psychiatry, NYU School of Medicine, NYU Langone Medical Center, New YorkNY, USA; ^2^The Emotional Brain Institute, The Nathan S. Kline Institute for Psychiatric Research, OrangeburgNY, USA

**Keywords:** piriform cortex, basolateral amygdala, odor coding, fear conditioning, top–down processing, optogenetics, channelrhodopsin

## Abstract

Olfactory information is synthesized within the olfactory cortex to provide not only an odor percept, but also a contextual significance that supports appropriate behavioral response to specific odor cues. The piriform cortex serves as a communication hub within this circuit by sharing reciprocal connectivity with higher processing regions, such as the lateral entorhinal cortex and amygdala. The functional significance of these descending inputs on piriform cortical processing of odorants is currently not well understood. We have employed optogenetic methods to selectively stimulate lateral and basolateral amygdala (BLA) afferent fibers innervating the posterior piriform cortex (pPCX) to quantify BLA modulation of pPCX odor-evoked activity. Single unit odor-evoked activity of anesthetized BLA-infected animals was significantly modulated compared with control animal recordings, with individual cells displaying either enhancement or suppression of odor-driven spiking. In addition, BLA activation induced a decorrelation of odor-evoked pPCX ensemble activity relative to odor alone. Together these results indicate a modulatory role in pPCX odor processing for the BLA complex. This interaction could contribute to learned changes in PCX activity following associative conditioning, as well as support alternate patterns of odor processing that are state-dependent.

## Introduction

Odor perception is strongly tied to emotion (Yeshurun and Sobel, 2010; Joussain et al., 2011; Kadohisa, 2013) and, in the extreme, olfactory perception has been suggested to be primarily a hedonic sense (Yeshurun and Sobel, 2010). The primary olfactory system has strong, reciprocal connections with regions known to be involved in encoding and recognition of emotional cues, including the amygdala, orbitofrontal cortex, and insular cortex (Cleland and Linster, 2003; Nigri et al., 2013). While odors can evoke unlearned hedonic responses, odors can also acquire emotional meaning through associative conditioning. The piriform cortex (PCX) a region important in encoding odor objects, and a direct target of inputs from hedonic associated regions, may play an important role in linking odor percepts to their emotional valence. The PCX is a paleocortical three-layered structure that directly receives odorant information relayed through the main olfactory bulb (OB). The PCX has been described as an associative cortex, with local connectivity between distributed pyramidal cells and between neurons of its anterior (aPCX) and posterior (pPCX) subdivisions (Haberly, 2001). This intracortical coupling allows for activation of distributed neuronal ensembles to represent the odorants being inhaled (Illig and Haberly, 2003; Rennaker et al., 2007; Stettler and Axel, 2009). The PCX is also broadly associative, as it shares reciprocal connectivity with a regional network including the orbitofrontal cortex, entorhinal cortex and amygdala, among other regions (Cleland and Linster, 2003). Due to this associative connectivity, we and others have hypothesized that odor-evoked activity within the PCX is not a simple representation of afferent input from the OB about inhaled odorants. Rather, PCX activity is also shaped by past experience, expectation, and current internal/behavioral state, conveyed to the PCX by this regional network. Evidence in support of associational and contextual processing in the PCX has been obtained in both human (Gottfried and Dolan, 2003; Karunanayaka et al., 2015) and non-human animals (Martin et al., 2006; Roesch et al., 2007; Chen et al., 2011; Gire et al., 2013). The strength and connectivity of the associative network is experience- (Kay and Freeman, 1998; Cohen et al., 2008; Cohen et al., 2015) and state-dependent (Hasselmo and Barkai, 1995; Linster and Hasselmo, 2001; Wilson and Sullivan, 2011), and shapes perceptual acuity and responses to learned odors (Chapuis et al., 2013). However, the mechanisms of regional network modulation of PCX odor coding are still only poorly understood.

The amygdala is an important component of this network, supporting associative memories and mediating adaptive behavior based, in part, on the emotional valence of sensory stimuli (Phelps and LeDoux, 2005). The amygdala is a necessary structure for behavioral odor-fear conditioning (Wallace and Rosen, 2001), and responds differentially to odor valence (Anderson et al., 2003; de Araujo et al., 2003; Moriceau et al., 2006). The amygdala receives odorant information in as few as two synapses from the nasal epithelium through direct connections from the OB to the cortical nucleus of the amygdala. The basolateral amygdala (BLA) has reciprocal connections with the PCX, with strongest connections to the pPCX (Majak et al., 2004). Glutamatergic axons from the BLA target both excitatory pyramidal cells and inhibitory interneurons in the PCX (Luna and Morozov, 2012), and are sufficient to drive EPSP’s in both cell classes. Given the diversity of target cell types, the consequences of BLA input on PCX odor responses may be complex. Here, as a first step in testing BLA modulation of PCX odor processing, we utilized optogenetic tools that enabled us to selectively stimulate BLA descending afferents in anesthetized mice while recording pPCX single-unit activity during odor stimulation. The results suggest a robust modulation of single-unit and ensemble odor-evoked activity following optogenetic activation of BLA afferents within the pPCX.

## Materials and Methods

### Mouse Subjects and Microinjection of Virus

Male and female C57BL/6J inbred adult mice were received from Jackson Labs (#664) and housed in the Nathan S. Kline Institute animal facility on *ad libitum* food and water at all times. All procedures were in accordance with NIH guidelines for the proper treatment of animals and the protocol was approved by the Nathan S. Kline Institute IACUC. 6- to 8-week old mice were used in survival surgery of microinjection of adeno-associated virus (AAV), as reported in Cetin et al. (2006) with adaptations. Specifically, mice were anesthetized with 1–4% isoflurane gas placed in a stereotaxic frame with heating pad and head-fixed with lambda and bregma level with one another. The mouse scalp was shaved, wiped with iodine, and treated with lidocaine local anesthetic gel before making an incision sagittally across the scalp surface to expose the skull. A single hole was drilled for craniotomy exposure of the brain surface at the site of injection using stereotaxic coordinates described below. A 5-μL calibrated micropipette (VWR #53432-706) was pulled to yield an elongated tip with a 20-μm diameter. A 0.4-μL suspension (≥1 × 10^12^ IU/mL) of AAV [AAV_5_-CaMKII-hChR2(E123T/T159C)-eYFP] was aspirated into the micropipette, which was then targeting to the LA/BLA using stereotaxic coordinates: -2.1 mm Bregma/3.05 mm Lateral/-4.0 mm Ventral from the meningeal surface. Five minutes after insertion of the micropipette, viral suspension was injected using a syringe pump at a volume flow rate of 50 nL/min. Each mouse was injected in one brain hemisphere only. After injection, the micropipette was left in place for 5 min. The micropipette was then raised 0.5 mm and left in place for an additional pause of 5 min before finally raising the micropipette slowly out of the brain. The scalp incision was then sutured together before treating the mouse subcutaneously with Buprenorphine (0.5 mg/kg) and intramuscularly with enrofloxacin (5 mg/kg) antibiotic. Mice recovered with an oxygen nose cone resting on a heating pad for 15 min before being returned to their cage, housed individually.

### Anesthetized *In vivo* Recordings

All recordings were made 3–4 weeks after AAV microinjection. Mice were anesthetized with urethane (1.25 g/kg) and placed in a stereotaxic apparatus. Respiration was monitored with a piezoelectric plethysmograph strapped to the animal’s back. A custom monopolar tungsten electrode was combined with 200 μm optical fiber to produce an integrated “optrode” that could both photostimulate BLA afferents in the pPCX and record local pPCX activity simultaneously. This reduced the possibility of indirect and unintended activation of alternate BLA input routes to the pPCX that may occur with direct photostimulation of BLA cell bodies, though did not completely eliminate the possibility of some antidromic BLA activation. To record from Layer II and III of the pPCX, the optrode was placed at stereotaxic coordinates: -2.0 mm Bregma/ 4.0 mm Lateral/ -2.9mm Ventral from meningeal surface and progressed to -3.3mm Ventral through multiple recordings in each mouse. All recordings of single unit activity and local field potential (LFP) were made relative to a scalp reference electrode. Signals were amplified and filtered (0.5–3000 Hz), digitized at 10 kHz, and stored and analyzed with Spike2 software (Cambridge Electronic Design Inc., Cambridge, England). Multiple protocols with varied combinations of odor and light stimulus were executed to analyze the relationship of BLA activation and pPCX odor processing. Odor-evoked single unit responses were analyzed as independent template-matched event waveforms using standard waveform analyses (Barnes et al., 2008; Chapuis and Wilson, 2012) A 473-nm laser (CrystaLaser #CL-473-100) was calibrated to deliver 5 mW at the optrode terminus before every recording. Photostimulation programs were universally delivered as 20 Hz trains of 10 ms light flashes for a 2-s duration. Temporal relationships between laser and odor stimulation were arranged in various combinations of “Before” (2 s laser train immediately preceding 2 s odor delivery, no overlap), “Overlap” (complete overlap of 2 s laser train and 2 s odor delivery), and “Delay” (Odor delivery for 2 s that is accompanied by a 2-s laser train that begins after 1 s of odor stimulus onset). Each program was repeated four times in sequential repeating order for every recording analyzed with at least a 60-s inter-stimulus interval to avoid odor habituation (Wilson, 1998a,b). Odor stimuli included variants of a 10-component (10c) mixture as previously described (Barnes et al., 2008; Chapuis and Wilson, 2012; Lovitz et al., 2012), with individual cells primarily tested with a single mixture. Mixture components were mixed in mineral oil to result in component concentrations (100 ppm for all components except 1,7-octadiene which was at 400 ppm) within the mixture on the basis of individual odorant vapor pressure. The 10c included the following monomolecular odorants (vapor pressure in mm Hg indicated in parentheses): isoamyl acetate (5.00), nonane (4.29), ethyl valerate (4.80), 5-methyl-2-hexanone (4.60), isopropylbenzene (4.58), 1-pentanol (6.11), 1,7-octadiene (22.1), 2-heptanone (3.86), heptanal (3.52), and 4-methyl-3-penten-2-one (6.69).

### Histology and Imaging

Immediately after recording, mice were intracardially flushed with 1× phosphate buffered saline (PBS) and then perfused with 4% paraformaldehyde in 1× PBS. Brains were then dissected and post-fixed further in 4% paraformaldehyde in 1× PBS overnight at 4°C. Brains were then rinsed in 1× PBS and then immersed in a 30% sucrose solution diluted in 1× PBS overnight at 4°C. Brains were then cryogenically mounted for microtome sectioning along the coronal plane at a 40-μM slice thickness. Sections were then arranged on glass slides and immediately mounted with coverglass and fluoromount-G (Southern Biotech #100-01) supplemented with DAPI (25 μg/mL) for nuclear counter-stain. Overexpression of the enhanced yellow fluorescent protein (eYFP) reporter fused to the channelrhodopsin construct utilized in our virally targeted transduction of LA/BLA tissue was sufficient to illuminate infected cells and their axonal fibers as early as one week after infection and up to four months as the latest time point examined. FITC-filtered epifluorescent microscopy was used to identify gross anatomical targeting of viral injection/infection. Successfully localized infections were then imaged using confocal microscopy to check both for axonal fibers expressing eYFP, as well as a lack of infected cell bodies in the PCX and endopiriform cortex that would confound the validity our optogenetic interrogation.

### Data Analysis and Statistics

Experimental group animals were organized based on localization of AAV infection as represented by eYFP expression. Mice that expressed the eYFP reporter selectively and exclusively in the LA/BLA were analyzed as the experimental group condition. Mice in the control condition on the other hand included recordings gathered from mistargeted injections showing eYFP reporter in other brain regions such as the dorsal hippocampus and motor cortex, which did not include the PCX, endopiriform, LA/BLA, medial amygdala, entorhinal cortex, nor any other structure monosynaptically connected to the PCX. All comparisons between experimental and control groups were statistically analyzed with an independent samples t-test of population means. Means were calculated as the absolute change in odor-evoked activity with laser stimulation of BLA afferents as compared to without (odor only stimulation). *In vivo* recordings analysis of single unit activity (Experimental *n* = 99, Control *n* = 65) were combined from multiple mice in each group (Experimental *n* = 17, Control *n* = 11).

## Results

**FIGURE 1:**
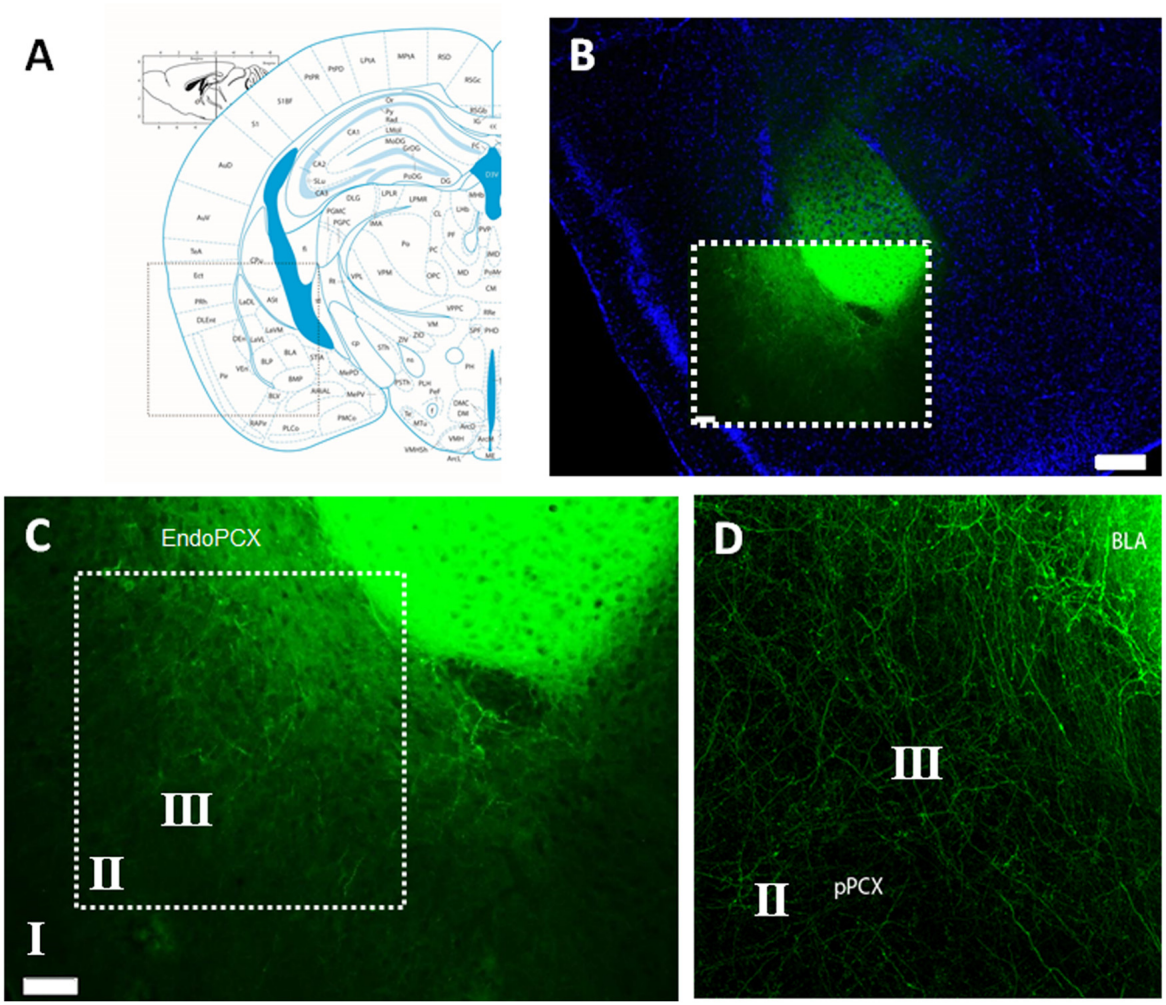
**Histological confirmation of localized adeno-associated virus (AAV) infection in experimental group animals recorded.** **(A)** Mouse brain atlas coronal view at -2.1mm Bregma with inset illuminated in **(B)** with nuclear counter stain of coronal brain cross section with infected lateral amygdala (LA) and basolateral amygdala (BLA), as visualized by the transduced Channelrhodpsin expression cassette with eYFP reporter fusion. **(C)** Magnified detail of inset from **(B)**, revealing the isolation of AAV infection, restricted to cell bodies of the LA/BLA and not cell populations inhabiting the piriform nor endopiriform cortex. **(D)** Confocal image with magnified detail of inset in **(C)**, showing infected LA/BLA afferent axon fibers innervating multiple layers of the piriform cortex (PCX). Scale bar in **(B)** = 500 μm, Scale bar in **(C)** = 100 μm. Roman numerals refer to cortical lamina.

**FIGURE 2:**
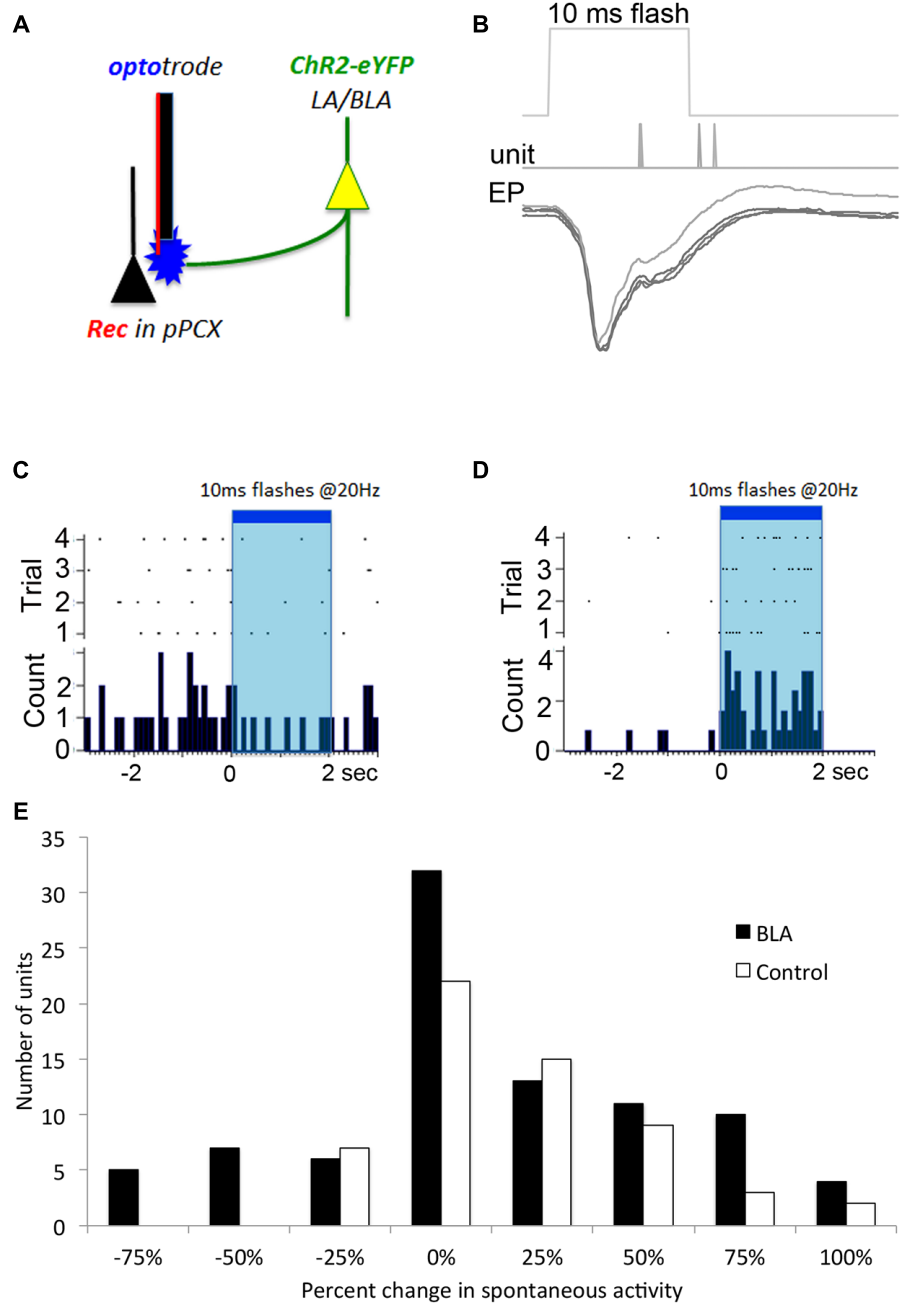
**Optogenetic *in vivo* approach used to test for BLA-piriform modulation.** **(A)** Diagram indicating the infected LA/BLA pyramidal cell populations that extend axons into the PCX. This enables photostimulation of BLA afferents locally at the PCX-recording site using an optrode. **(B)** Local field potential response recorded *in vivo* after single 10 ms pulse photostimulation is delivered via depth optrode as in **(A)** in a BLA-infected animal. Repeated stimulations are over-laid, and time of evoked spiking is also displayed. These responses are characteristic of monosynaptic activity. Single unit monitoring of different example cells’ spontaneous activity levels were seen to be both **(C)** suppressed and **(D)** excited in response to BLA photostimulation. **(E)** Frequency distribution of BLA fiber photostimulation effects on pPCX single-unit spontaneous activity in BLA-infected and control animals.

To specifically activate BLA afferent inputs to the pPCX, we performed stereotaxic microinjections of AAV carrying a channelrhodopsin construct [ChR2(E123T/T159C)] directly into cell bodies of the BLA. This overexpression cassette is driven by the CaMKII promoter and is therefore preferentially expressed in excitatory pyramidal cells. Additionally, optrode placement for all recordings was directed to the pPCX, thereby isolating infected neuron photoactivation to afferent fibers innervating the pPCX from infected BLA cell bodies (**Figure 1A**). Anatomical tracing studies have revealed direct synaptic contact from both lateral and BLA axon fibers to all three layers of the pPCX, with layers II and III most heavily innervated (Majak et al., 2004). Viral transduction and construct expression were given 3–4 weeks post-injection to accumulate to maximal levels of ChR2 protein at axon terminal surfaces. Immediately after recording, mice were fixed and brains sectioned to check the fusion construct eYFP reporter for accuracy of viral targeting and isolation of infection (**Figure 1B**). Confocal imaging allowed us to confirm that only axon fibers were targeted and that this did not include infected cell bodies in the pPCX cell layers nor the endopiriform nucleus (**Figures 1C,D**), as these outcomes may confound our photostimulation analysis intended for BLA afferents alone (**Figure 2A**).

To confirm that BLA infection with the ChR2 construct resulted in photoinducible activity in local pPCX circuitry, we performed anesthetized recordings in the pPCX of infected animals, using an optrode for simultaneous photostimulation and recording at the same terminus. Laser pulses (0.5–10 ms) resulted in reliable, short latency-evoked field responses (**Figure 2B**). No response was observed when the fiber optic connection to the laser was removed. Furthermore, photoactivation was specific to infected tissue and was not an artifact of laser energy at the optrode terminal, as non-infected controls showed no evoked potential to light stimulation (data not shown). Direct modulation of spontaneous single unit activity could also be detected in infected animals, with both excitatory and suppression effects observed in different cells during extended laser pulse trains (**Figures 2C,D**), with a trend toward more cells showing excitation than suppression (**Figure 2E**). Analysis of the proportion of cells showing a greater than 50% change in spontaneous activity induced by light showed significantly more cells changed their activity in the BLA infected group than the control group (χ^2^ = 7.58, *p* < 0.01). It should be noted that the magnitude or lack of effect of photostimulation on a given cell’s activity may reflect either a real variation in responsiveness to BLA input, or variation in infected terminals directly or indirectly modulating that cell. Nonetheless, these results demonstrate BLA modulation of pPCX single-unit activity *in vivo*.

**FIGURE 3:**
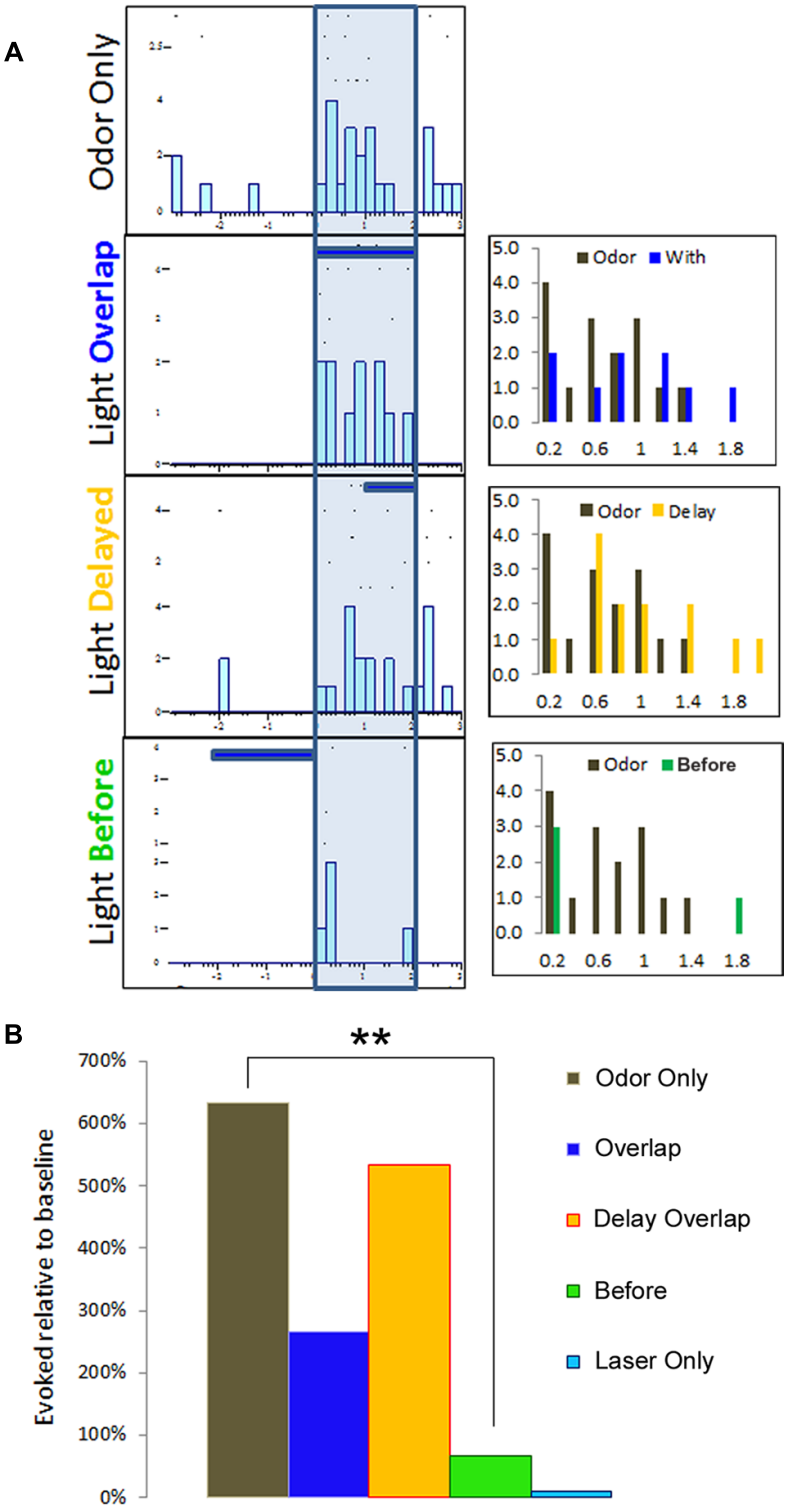
**Multiple temporal relationships of odor and BLA photostimulation were examined for single-unit recording purposes.** In this representative example, **(A)** raster plots summarizing a single-unit response to three different protocols of odor and light optrode delivery. The three histograms to the right represent each condition comparison to the odor-only baseline activity, organized into 200 ms time bins. **(B)** Single-unit activity from **(A)** is summarized here to indicate the protocol with a BLA-photostimulation train preceding odor delivery resulted in the most dramatic modulatory effect, as compared to either overlapping photostimulation and odor or delayed photostimulation overlap with odor delivery. ^∗∗^*p* < 0.01, repeat measures ANOVA comparing mean number of spikes per 200 ms time bin during 2 s odor delivery epoch between light-odor protocols and odor-only.

To monitor for modulatory influence of BLA activation on pPCX odor responses, we delivered odor mixtures to anaesthetized mice and recorded single-unit responses in the pPCX. We tested three protocols that combined both odor delivery and photostimulation in varying temporal relationships: (i) a 2-s train of 10 ms laser flashes at 20 Hz completely overlapping in time with a 2-s odor delivery, (ii) a laser “delay” arrangement, with a 2-s odor delivery accompanied 1s after onset by the same 2-s laser train, and (iii) a laser “before” arrangement, with the 2-s laser train immediately preceding a 2-s odor delivery (**Figure 3A**). Single-unit odor-evoked activity was quantified by subtracting baseline firing rates (pre-odor and pre-laser as appropriate). Total cumulative odor-evoked single unit activity (per 200 ms bin) within each recording was quantified as event counts within each 200 ms time bin typo, singular during the 2-s odor delivery epoch. Cumulative evoked spike counts in each condition were statistically compared to the odor only condition in the same cell. The most powerful and consistent modulation effect across cells was obtained with the laser before odor protocol, as determined by one-way ANOVA across conditions within each recording (example one-way ANOVA of the recording results illustrated in **Figure 3A**: *F*_[3,39]_ = 3.78, *p* = 0.022, Tukey’s posthoc test [Odor Only vs. Laser Before Odor] *p* < 0.05, other post-test pairs not significant, **Figure 3B**). Due to its superior effect measured, the remainder of the data presented here utilized the laser-before photostimulation protocol.

**FIGURE 4:**
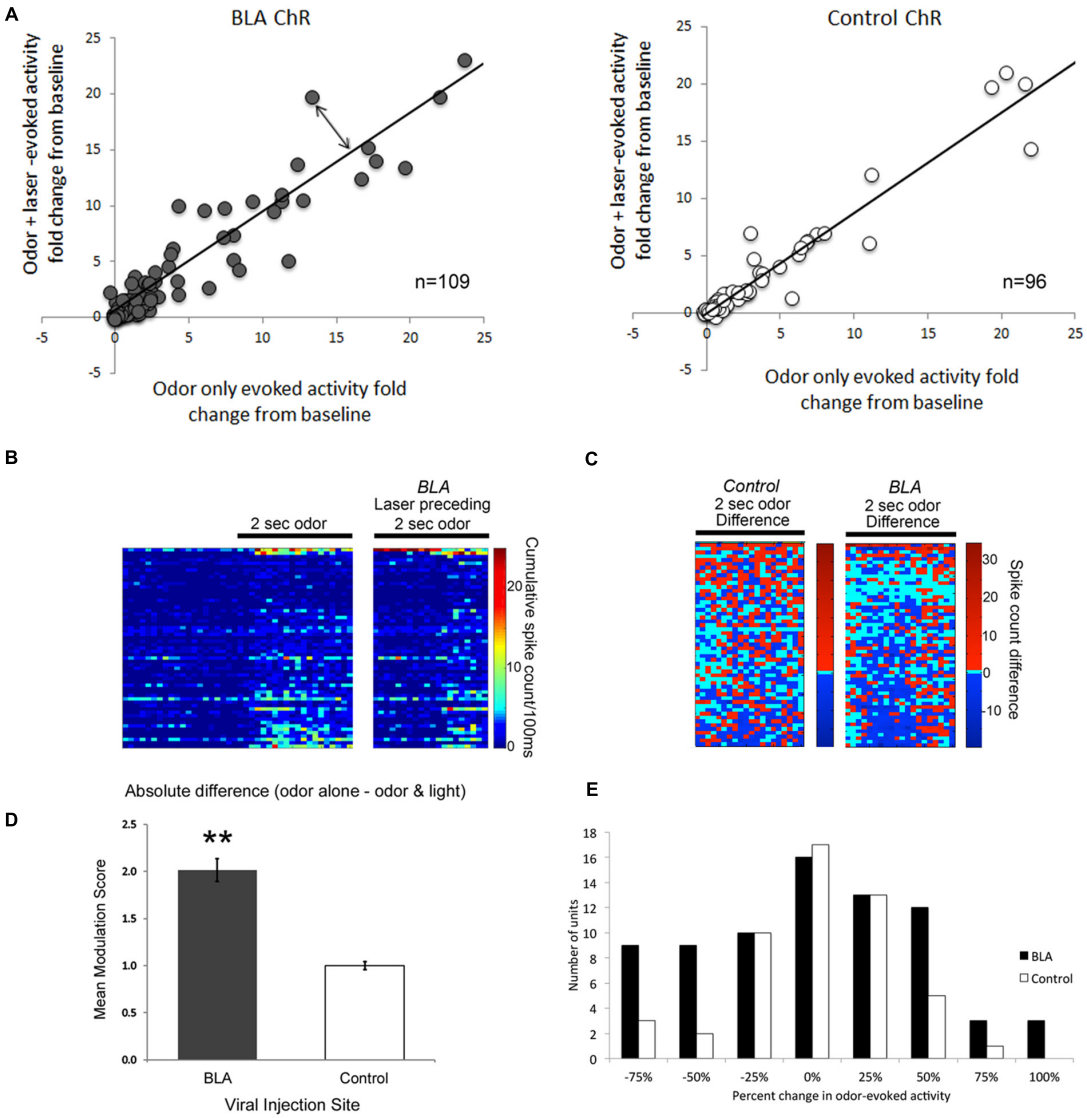
**A Comparison of single-unit responses to odor delivery with and without preceding BLA-photostimulation.** **(A)** Scatterplots of both BLA-infected (grey) and non-BLA infected (white) control animal recordings of single-unit activity. The fold change in single unit odor-evoked activity was plotted in relation to odor response when preceded by BLA-photostimulation. The line represents a perfect correlation that indicates little to no difference in effect as a result of BLA photostimulation. The further each datapoint lies from the line (double-ended arrow) indicates a more dramatic modulation effect. Only those cells with smaller than a 25-fold change are plotted for clarity, though all cells are included in statistical analyses. **(B)** Pseudocolor rasterplots of experimental single-unit cumulative activity in response to odor only (left), odor preceded by BLA fiber photostimulation (right). **(C)** The difference between these two conditions for BLA-infected animals (right) and controls (left). For **(B)** and **(C)**, each row across all three plots is a different single-unit and units have been sorted by the magnitude of their activity difference in **(C)**. The two plots are matched for overall size. **(D)** A summary of data in **(A)**, represented as the mean modulation index calculated as the absolute fold change in single-unit activity resulting from preceding BLA-photostimulation. ^∗∗^*p* < 0.01, independent samples *t*-test. **(E)** Frequency histogram of change in odor-evoked activity (summed over the 2-s stimulus) in the BLA fiber stimulation condition relative to the odor only condition in BLA-infected and control animals. The most common large effects were in the suppressive direction.

Single-unit responses to odor were significantly modulated by photoactivation of BLA fibers prior to odor onset, though the direction of the effect varied between cells. Some cells showed reliable enhancement of odor-evoked activity by pre-activation of BLA fibers, while other cells showed reliable suppression (**Figure 4**). As shown in the rasterplots of difference between odor only and odor + BLA fiber activation (**Figure 4C**) and the frequency histogram of effect sizes (activity differences summed over the entire 2 s odor; **Figure 4E**), the largest effects were in the suppressive direction. To statistically compare light modulation of pPCX single-unit odor-evoked activity in experimental BLA-infected animals vs. non-BLA-infected control animals, individual unit recordings were pooled and compared between conditions (**Figure 4**). A modulation index was calculated for each single-unit as the absolute value (odor-only-evoked spike counts – odor with laser-evoked spike counts). A modulation score of 0 signifies no difference between the odor only and odor + laser conditions. Individual data are plotted in **Figure 4**. The mean modulation index for experimental BLA pre-stimulated units was significantly greater than uninfected BLA control cells (**Figure 4D**; Independent samples *t*-test: *t*_204_ = 3.05, *p* = 0.0026). A further analysis of the proportion of cells showing a greater than 50% change in odor-evoked activity induced by light showed significantly more cells changed their odor-evoked activity in the BLA-infected group than the control group (χ^2^ = 4.89, *p* < 0.05). Interestingly, there was no significant correlation between single-unit response to the laser alone (spontaneous activity) and how the laser modulated odor-evoked activity (*r* = 0.06).

**FIGURE 5:**
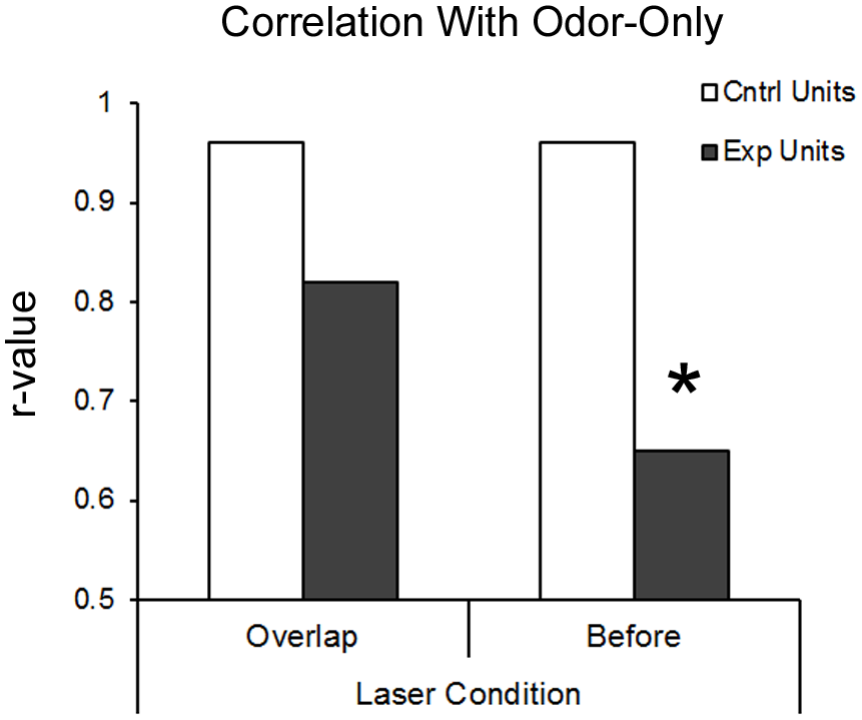
**BLA-stimulation results in decorrelation of posterior piriform single-unit ensemble responsive to odors in BLA-infected animals.** Both protocols of BLA-photostimulation before-odor and light-odor overlapping resulted in lower *r*-values for BLA-infected animals compared to the same treatments in non-BLA infected animals. However, only BLA-photostimulation before odor protocol resulted in significant deccorelation, as determined by *r*-value to *z*-stat conversion. ^∗^*p* < 0.01.

An additional metric of BLA modulation of pPCX odor response combined all recorded cells into a pseudo-ensemble. We asked whether the activated of BLA fibers induced a decorrelation of ensemble odor-evoked activity relative to odor presentation alone. If for example BLA input simply elevated odor-evoked firing rates of all cells equally, the ensemble response to odor + laser would be highly correlated with the ensemble response to odor alone. If however, different cells within the ensemble were affected differently, a decorrelation should emerge between the two stimuli. As shown in **Figure 5**, in control cells, ensemble activity in response to odor-only was highly correlated with odor + laser (*r* = 0.96). However, in BLA targeted animals, BLA input activation induced a decorrelation between odor only and odor + laser (*r* = 0.58; significantly different from control, *r* to *z* transform, *z* = 7.33, *p* < 0.01), suggesting a differential encoding of odors experienced in combination with BLA activity compared to odors experienced in the absence of BLA activity. As noted above, this decorrelation was most pronounced with BLA activation occuring immediately prior to odor onset.

## Discussion

The present results demonstrate robust modulation of pPCX odor-evoked activity by BLA input. There was diversity in single-unit response to BLA optogenetic activation, as well as diversity in BLA modulation of odor responses. Thus, a subset of pPCX neurons were excited by activation of BLA fibers, a subset was unresponsive, and a subset showed suppression of spontaneous activity. A similar broad diversity was demonstrated in how BLA fiber activation influenced single-unit odor responses. This modulation however, was significantly greater than light induced changes in animals that had AAV-ChR injections that did not target the BLA. In addition to BLA induced changes in single-unit response to odor, BLA fiber activation also modified pseudo-ensemble responses to odors, suggesting a modification of population coding of pPCX odor coding in the presence of BLA input. This decorrelation suggests that BLA activity may not simply act as gain control over the pPCX, but actually modify the encoding of the odor itself, perhaps influencing perceptual odor quality. This modification may underlie the unique response to learned aversive odors observed in PCX (Moriceau et al., 2006; Sevelinges et al., 2008; Chen et al., 2011; Choi et al., 2011), and contribute to learning and memory of odor fear.

The diversity in single-unit responses to BLA input, even within animals, most likely reflects a diversity in target cell innervation by infected BLA fibers and diversity of cell types recorded here. Thus, some, if not all of the cells showing no BLA modulation may have not been targeted, directly or indirectly by BLA AAV infected fibers. The lack of effect in these cells must therefore be considered with caution. Regarding diversity in cell types, it has been demonstrated that BLA input to PCX targets both interneurons and pyramidal cells in PCX (Luna and Morozov, 2012), thus a given neuron’s response to odor+laser would reflect a complex local circuit output. Furthermore, these data can not rule out antidromic activation of BLA neurons by light stimulation of fiber terminals. Thus, some effects seen here could result from indirect effects of BLA stimulation. Nonetheless, both scenarios – direct effects of BLA terminal activation within the PCX, and indirect effects of BLA activation – result in the same conclusion that BLA activity modulates odor coding in PCX. Future research, recording odor responses from identified cell types is required to further characterize this relationship.

It should also be emphasized that of the stimulation protocols examined here, activation of BLA fibers immediately prior to odor onset, rather than during odor stimulation, evoked the most robust changes in single-unit odor-evoked activity. This suggests that perhaps initial pPCX circuit conditions, at odor onset, are most critical for modulating odor responses in this way. It further suggests a relatively long-lasting (seconds) modulation of pPCX circuit activity following BLA activation. A similar long-lasting effect of amygdala activity on sensory coding has been demonstrated in the primary auditory cortex (Armony et al., 1998). Single-units in the primary auditory cortex show enhanced, prolonged responses to a fear conditioned CS+, with for example, maintained enhanced activity for the duration of a 2-s tone. Amygdala lesions have no effect on the initial enhanced response to the tone (0–50 ms), but completely blocked the prolonged response (500–1500 ms; Armony et al., 1998). The importance of amygdala activity for sensory cortical-evoked activity over 1–2 s matches well with that observed here in the pPCX. Furthermore, the observed temporal sensitivity may also reflect the fact that the current recordings were performed in anesthetized animals. Future work will require analysis of these effects in awake animals, perhaps performing an odor-guided task.

These results suggest a mechanism for the observed changes in PCX activity to learned aversive odors observed with a variety of assays (Moriceau et al., 2006; Sevelinges et al., 2008; Chen et al., 2011). For example, previous work from our lab examining PCX single-units in awake animals undergoing different kinds of odor aversion training showed task-specific changes in PCX activity that correlated with learned odor fear (Chen et al., 2011). Thus, using a differential conditioning paradigm that involved both a CS+ and CS-, animals learned highly specific odor fear, and PCX single-units expressed narrowed odor receptive fields. In contrast, animals trained without the CS- learned generalized odor fear, and PCX single-units showed broadened odor receptive fields. The present data suggest that activation of BLA neurons during conditioning may have contributed to the emergence or expression of these different PCX odor coding outcomes. The fear conditioning-induced changes were recorded in the aPCX, while the recordings here were in pPCX. The BLA targets both of these structures, though with more dense projections to pPCX (Majak et al., 2004). Given this difference in anatomy and the previous *in vitro* work focusing on pPCX (Luna and Morozov, 2012), we chose to record from pPCX here. Nonetheless, we predict similar direct or indirect BLA modulation in aPCX. A similar process is believed to occur with auditory fear conditioning, wherein amygdala projections to the auditory cortex differentially modulate single-unit responses to the tone conditioned stimulus (Armony et al., 1998). Further characterization of this pathway, including plasticity is ongoing.

Finally, the results emphasize that understanding odor processing in the PCX must not only take into account the spatiotemporal patterns of OB input, it must also take into account the context of inputs from regions such orbitofrontal cortex (Illig, 2005; Cohen et al., 2015), entorhinal cortex (Faillace et al., 2006; Chapuis et al., 2013) and amygdala (Majak et al., 2004; Luna and Morozov, 2012) into which that OB input flows. PCX connectivity with this broader context is both state- (Kay and Freeman, 1998; Wilson and Yan, 2010) and experience-dependent (Cohen et al., 2008, 2015; Chapuis et al., 2009), suggesting a basis for dynamic cortical odor processing. Interestingly, our results may be related to effects of amygdalar modulation of odor processing in human studies. Induction of anxiety results in a shift in valence evaluation of neutral odors in a negative direction (Krusemark et al., 2013). This shift in perceptual reference coincides with increased activity in the amygdala and a significant decrease in posterior piriform activity, indicating a modulatory effect similar to that observed here.

## Author Contributions

BS collected the data, analyzed the data and helped prepare the figures and manuscript. DW analyzed the data and helped prepared the figures and manuscript.

## Conflict of Interest Statement

The authors declare that the research was conducted in the absence of any commercial or financial relationships that could be construed as a potential conflict of interest.
